# CRISPR-Cas9 library screening approach for anti-cancer drug discovery: overview and perspectives

**DOI:** 10.7150/thno.71144

**Published:** 2022-04-11

**Authors:** Yau-Tuen Chan, Yuanjun Lu, Junyu Wu, Cheng Zhang, Hor-Yue Tan, Zhao-xiang Bian, Ning Wang, Yibin Feng

**Affiliations:** 1 School of Chinese Medicine, The University of Hong Kong.; 2 School of Chinese Medicine, Hong Kong Baptist University.

**Keywords:** CRISPR-Cas9, Library screening, Cancer therapy, Drug discovery, Experimental models

## Abstract

CRISPR-Cas9 is a Nobel Prize-winning robust gene-editing tool developed in the last decade. This technique enables a stable genetic engineering method with high precision on the genomes of all organisms. The latest advances in the technology include a genome library screening approach, which can detect survival-essential and drug resistance genes via gain or loss of function. The versatile machinery allows genomic screening for gene activation or inhibition, and targets non-coding sequences, such as promoters, miRNAs, and lncRNAs. In this review, we introduce the emerging high-throughput CRISPR-Cas9 library genome screening technology and its working principles to detect survival and drug resistance genes through positive and negative selection. The technology is compared with other existing approaches while focusing on the advantages of its variable applications in anti-cancer drug discovery, including functions and target identification, non-coding RNA information, actions of small molecules, and drug target discoveries. The combination of the CRISPR-Cas9 system with multi-omic platforms represents a dynamic field expected to advance anti-cancer drug discovery and precision medicine in the clinic.

## Background

The Clustered Regularly Interspaced Short Palindromic Repeats (CRISPR)-associated endonuclease 9 (Cas9) protein was first reported by two Nobel laureates, Charpentier and Doudna, in 2012. It was initially a natural immunity weapon against viral infections in *Streptococcus sp.* The researchers invented the target DNA cleavage mechanism by integrating CRISPR RNAs (crRNAs) with manually designed trans-activating crRNA (tracrRNA) to form single-guide RNAs (sgRNAs) 1. Endonuclease Cas9 protein is guided to the target site acting as “scissors” to cleave the DNA, leaving either a double-strand break (DSB), single-strand nick, or mutagenesis 2, 3. Previous gene-editing technologies, like zinc-finger nucleases (ZFNs) and transcription activator-like effector nucleases (TALENs), require customizable, specific DNA sequence-binding modules fused to the non-specific DNA endonuclease domain. It was advantageous to engineer targeting proteins for editing any DNA sequence with new designs for target sites. Nevertheless, this complex cloning for customized proteins is difficult to be scaled up for whole-genome applications.

Before the advent of CRISPR-Cas9 pooled libraries, RNA interference (RNAi) screens have been widely used. The most applied RNAi pathway is the short hairpin RNAs (shRNAs), which inhibit post-transcriptional levels of mRNAs by inducing endogenous interference via the RNA-induced silencing complex (RISC) 4. Since RISC acts on cytoplasmic RNAs, it does not affect cell ploidy, DNA tertiary structure, and chromatin conformation. The transduction process is more straightforward than the CRISPR-Cas9 system, as no exogenous sequences coding for endonucleases and transcriptional modifiers are involved. Despite the practical applications of RNAi in many complicated cellular models, its performance in general library screens, especially cancer-related models, has not been satisfactory. The knockdown efficiency is usually unstable and incomplete, making the quality control of the library screens challenging. This problem is most evident in the survival-essential genes 5. Also, there are reports of persistent off-target activity, minimizing the significance of the screening results 6. The action of the RNAi machinery is also limited, mainly inside the cytoplasm 7. Due to its suboptimal efficiency and off-target effect, many shRNAs are usually designed to target a single gene. The nature of shRNAs makes carrying unique barcodes on each backbone impossible. Extra steps are needed to analyze sequencing results after the screening 8. Hence, more promising library screening approaches are needed, and the CRISPR-Cas9 platform is an excellent tool to fill the gap.

The CRISPR, unlike its ancestors, utilizes a universal Cas9 protein that needs only a guided RNA (gRNA) to match the target. This convenient, rapid, but versatile genome editing technology provides a promising library screen application to identify essential genes for cell survival and drug resistance 9-12 (Table 1). The introduction of the CRISPR-Cas9 system into the library screen application has primarily enhanced the utilization potential of the genome-editing tool. This review focuses on how the genome editing function of CRISPR-Cas9 could be adapted to screen for genes of interest with different experimental model designs in cancer research. We discuss various applications of the pooled library screening approach in anti-cancer drug discovery and the advantages and limitations compared with other current technologies. We also present the latest development of the CRISPR-Cas9 library screening with other omic platforms to provide insights to researchers in this field.

## Varieties of CRISPR-Cas9 Pooled Libraries

The application of the CRISPR-Cas9 as a gene-editing tool in different research fields has been widely studied, and several reviews on its fundamental principles, variations, and applications in cancer research have been published 13-17. The pooled library screening approach, using CRISPR-Cas9 based gene-editing tool, has evolved as a powerful way to identify interesting gene mutations through phenotypic changes or viability screens. Currently, customed-designed CRISPR-Cas9 libraries covering the whole genome for essential gene screening or a series of genes with specific phenotypes or cellular functions for target discovery are employed. The experiments are designed not to perturb each cell more than once. The unique genetic change is typically ensured using a viral-packaged sgRNA library at a less than 0.3 to 0.5 low multiplicity of infection (MOI) 18. In the case of combinatorial screens, gene alterations are performed in multiple (usually two) turns to evaluate combined gene functions 19, 20.

Cas9 variants have been engineered to adopt different screening strategies to cope with experimental needs and settings. Typical Cas9, which brings about gene knockout (CRISPRko) by inducing error-prone non-homologous end joining (NHEJ) DNA repair or error-free homology-directed repair (HDR), results in irreversible KO indels. In recent developments, variations of Cas9 proteins have emerged (Table 1). Utilizing the Cas9 mutant nickase version (Cas9n) to create nicking of both DNA strands separately by a pair of gRNAs, the genome-editing results in site-specific DSB 21. This approach maximizes the specificity while maintaining efficiency similar to wild-type Cas9 22. The use of catalytically inactive Cas9 (dCas9) could repress target genes by transcriptional inhibition, also known as CRISPR interference (CRISPRi) 23-25. The dCas9 protein combined with transcriptional repressors, such as KRAB, instead of DNA cleavage, binds to and hinders the target region in the genome, suppressing the RNA polymerase activity and leading to gene repression 1. On the other hand, the fusion of effector domains e.g. VP64 26, enables reversible transcriptional activation 18, 24. The Cas9 complex is guided by sgRNAs but binds to the promoter region of the gene of interest. Another advancement in Cas9 technology is adding a base editor to dCas9 or Cas9n. Deaminase enzymes or adenine base editors (ABEs) can introduce point mutations and enable nucleotide conversion in target sites 27, 28. After these phenotypic screens, total RNA is extracted, sequenced, and relative levels of all sgRNAs in control and experimental samples are analyzed 18. Subsequently, the genes of interest are identified by negative or positive selection.

### CRISPRko

**Figure 1:**
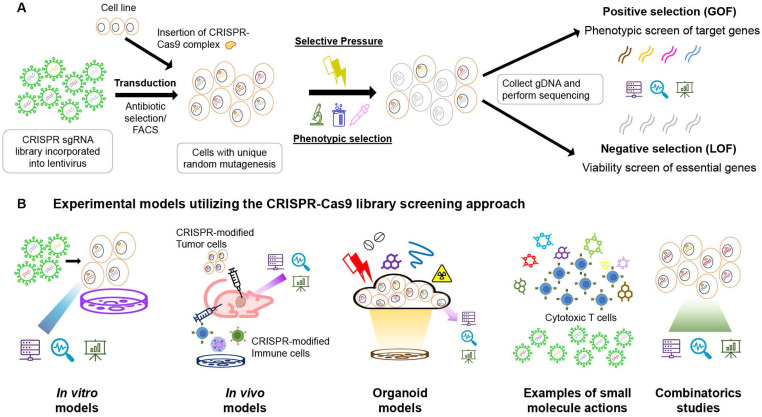
** (A)** Schematic diagram of the CRISPR-Cas9 library positive and negative selection workflow. The sgRNA library is incorporated into the lentivirus. The library is then transduced into the cell line (with CRISPR-Cas9 complex inserted in the case of the two-plasmid system, or an all-in-one backbone packed inside the lentivirus) with an appropriate MOI (usually less than 0.3-0.5) to ensure unique mutagenesis per cell. The transduced cells were undergone antibiotic selection or FACS to select for successful modifications. The remaining cells are then put under selective pressure or phenotypic selection for desired cell functions. The gDNA of the survived cells are extracted and then subjected to next-generation sequencing to obtain information on the sgRNA presence. The existing amount of sgRNAs are ranked from highest to lowest, and the positive selection is from the top, while the negative selection is from the bottom. **(B)** Experimental models utilizing the CRISPR-Cas9 library screening approach for anti-cancer drug discovery including *in vitro* models, *in vivo* models, organoid models, combinatorics studies and small molecules screenings. In *in vitro* models, sgRNA libraries are introduced into the cell pool by viral means (usually lentivirus), while the sgRNAs and Cas9 protein complex could be transduced in single or multiple vectors. The foreign genetic materials are often expressed using eukaryotic transposons. In *in vivo* models searching for target genes in tumours, the cell lines are usually modified in the culture and then injected into animal bodies to form tumour masses. After drug treatments or survival studies, genomic DNAs will be extracted from the tumours and analyzed for the sgRNA appearances using deep sequencing. In some cases, with no handy orthotropic model available, or limited patient-derived xenografts, cultured organoids are ideal models to study for the tissual response of the tumor to the drugs. Selective pressure is applied on the organoid culture with CRISPR-Cas9 library integrated, and the remaining bodies are allowed to grow, and gene mutation pattern is analyzed afterwards. Small molecules are emerging research directions in the study of cancer drug resistance. Large-scale drug screening is comprehended with a CRISPR-Cas9 library screen to detect the potential molecular candidates and genes responsible for drug resistance on the CAR-T therapy. The screening result could be analyzed on both GOF and LOF to look for mutations leading to drug resistance genes and damaged essential proteins. In combinatoric studies, two independent genetic modifications are induced by two sgRNAs. The cells are either exposed to the lentivirus twice for second modifications, or integrating a dual-Cas9 enzyme system with two independent target sites in single transduction. It is possible to induce a knockout and overexpression in the same run simultaneously.

Compared with the controls, the negative selection screens identify the depleted or reduced levels of sgRNAs in the population. CRISPRko is often used to detect the loss of fitness in the population, such as reduced viability, drug sensitivity, cell proliferation, and incapability of migration (Figure 1). The sgRNA targets could be designed for nearly all genome regions and not just the functional genes, and the non-coding components, such as promoters, enhancers, and miRNAs, could be modulated directly 9, 29, 30. Pooled screens utilizing typical CRISPR-Cas9 guided by sgRNAs resulted in indels by NHEJs 31, convenient for detecting survival-essential genes or fitness genes with increased sensitivity compared to the previous RNAi platforms 5, 31.

However, the CRISPRko system has low cutting efficiency and off-target effect limitations. The native Cas9 system requires more (around 10) sgRNAs on each target to ensure the effective knockout of a specific gene. Also, despite well-designed sgRNA libraries that could minimize off-target effects 5, 32-34, heterogeneous or heterozygote indels and knockouts remain a concern. When specific mutations fail to induce a stop codon or frameshift, the efficiency is reduced. The sgRNA potency is essential to target the biallelic functional gene mutations 32. The lowered achievable depletion screening ability could be remedied by sequence-specific barcodes 33, 34. Intriguingly, the introduction of DSBs on the genome also elevated cell toxicity and jeopardized cell fitness, leading to false-positive selections, which further hinder their applications in combinatorial screens with multiple targets per cell 35, 36.

### CRISPRi

The CRISPRi systems integrated with dCas9 reversibly knockdown target genes without perturbating the genome sequence. The loss-of-function screening can be adopted without causing any unpredictable non-target cell toxicity. The sgRNA targets are designed from 50 base pairs before to 300 base pairs after the transcription start site (TSS) with the protospacer length between 18 to 21 base pairs 37. Homopolymers in the sequence that could substantially hamper the sgRNA activity should be avoided 38. Since dCas9 directly acts on the homogeneous TSS, and by hindering 23 base pairs on the target genome only 39, CRISPRi could interfere precisely with regulatory elements and non-coding RNAs, including miRNAs and lncRNAs 40, 41. Especially, lncRNAs could not be easily knocked down with RNAi or CRISPRko as their gene functions are usually silenced by a consequential mutation or multiple indels 10, 42, 43. The RNAi also has limited efficacy in inhibiting the lncRNAs localized in the nuclei 7.

The CRISPRi system is usually adopted with the most common extra-regulatory domain, the Krüppel-associated box (KRAB), a DNA-binding-dependent transcriptional repressor from the amino terminus of the zinc finger protein 10 44. A more recent example of the CRISPRi setup is a dCas9 protein fused with the C-terminal gene-silencing effector domain, KRAB-MeCP2, 45. This gene suppressor matrix had a more potent effect on most single or multiplex sgRNA targets than the gold standard repressor, dCas9-KRAB. The MeCP2 domain binds to different transcriptional regulators, including DNA methyltransferase and SIN3A-histone deacetylase corepressor complex, further suppressing the target genes. However, the high sensitivity of CRISPRi makes it more vulnerable to sequence variability, for instance, DNA polymorphism in regulatory regions 37. Furthermore, as the CRISPRi functions by binding to the TSS, this may not be a perfect choice for genes regulated by more than one TSS or multiple genes regulated by a single TSS 36.

### CRISPRa

The gain-of-function (GOF) screens are analyzed by positive selection, where the most enriched targets constitute the genes of interest (Figure 1). Before the CRISPRa platform, GOF screens were usually carried out by cDNA library overexpression, which had many drawbacks, such as incomplete coverage, difficulties in design and construction, and lack of endogenous regulations and variability 46, 47. On the contrary, CRISPRa activates gene expression by targeting promoter regions of the corresponding loci. Based on the targets, only sgRNA libraries are needed and gene transcription is activated endogenously on the proximal promoters. The design and cloning of sgRNAs are easy and inexpensive than previous approaches. The typical CRISPRa involves a Cas9 or dCas9 protein integrated with a transcriptional activator. Tanenbaum *et al.* employed the SunTag peptide 48, which is a multiple peptide array of epitopes with variable antibody fragments, to recruit VP64 effector protein domains 37. The VP64-p65-Rta complex, when fused with the dCas9 protein, could also act as a transcriptional activator on endogenous coding and non-coding genes 49. Another platform developed by Zhang's group incorporated the MS2 sequence onto the sgRNA backbones to recruit two more effector domains, p65 and HSF1. This synergistic activation mediator (SAM) complex, together with the dCas9-VP64 fusion protein, could overexpress the target gene by upregulating transcription. Zalatan *et al.* integrated CRISPR sgRNAs with scaffolding RNA sequences to allow multidirectional regulations by recruiting protein effectors and epigenetic modifiers into the target region 20. Various levels of regulation could be achieved by implementing different regulators.

### Point Mutagenesis

Besides indel mutations or DSBs leading to frameshifts or nonsense mutations, Cas9n is the emerging version of the CRISPR genetic editing system. Since many genetic diseases arise from point mutations or single-nucleotide polymorphisms, it is necessary to develop a tool that could precisely make single base pair changes. Current CRISPR-Cas9 systems integrated with base editing usually consist of deaminase activity. The cytidine deaminase enzyme can mediate C to T or G to A conversion without causing DSBs or frameshifts. For instance, rat cytidine deaminase enzyme APOBEC1 with a potent conversion activity, when fused with a 16-residue XTEN linker, could extend its effective action range to 4-8 base pairs from the distal end of the protospacer 27. The efficiency of base editing was higher than 50%. Only 4-6% indels were observed at the target sites in astrocytes compared to the 26-40% indels with the wild-type Cas9 and no evident base repair. However, Cas9n is not yet commonly applied in library screens. Among the few other options available for generating base pair changes, mutation locations could only be determined by site but not the precise base 50. The mutation frequencies of different bases within the mutation window are different and might be biased when applied to a genome-wide screen 51. Also, the base editing protein complex is substantial, requiring efficient intracellular delivery tools, and its function may be impaired in some dense genome structures 52.

## CRISPR-Cas9 Experimental Models for the Discovery and Development of Anti-cancer Drugs

The CRISPR library screening method is one of the most adaptable research methods for forward candidate gene identification from the phenotype-to-genotype approach. Various applications of CRISPR-Cas9 library screens for the anti-cancer drug target discovery are discussed below (Figure 1) (Table 1).

### *In vitro* Models

The CRISPR system from the bacteria was adapted as an effective genetic editing tool in 2013, and subsequently, the first genetic screens using the CRISPR-Cas9 system were reported 9-12. Cell lines stably expressing the Cas9 protein were established, and vectors coding the sgRNA were delivered through lentivirus. Also, a different method integrating the Cas9 protein-coding sequence with the corresponding individual sgRNA segment into the same lentiviral backbone was chosen by Shalem *et al.* 9. The single lentiviral vector carrying the Cas9 protein, sgRNA, and the selection marker makes this platform more accessible to any cell line of interest without establishing the Cas9-expressing cell line. Koike-Yusa *et al.* used the piggyBac transposon to carry and express Cas9 11, while promoters like doxycycline 10 and OCT1 12 were also employed. The efficiency of the null mutagenesis was greatly enhanced when sgRNAs were targeted directly into the coding exons of important protein domains 53.

### *In vivo* Models

*In vivo* models are established to study the effect of target genes in the tumor tissue or on the microenvironment, facilitate screening effectiveness, and study the primary tumor growth or metastasis. The most common method to establish a tumor model with library screens is by culturing the engineered cells *in vitro* and then transplanting them into the animals 30. For instance, the NSCLC cell line KPD was transduced with the Cas9 protein and a GFP marker to ensure the homogeneity of the cell line and assist in monitoring tumorigenesis and metastasis. The library-transduced cells were injected into the mice after culturing for one week. Genomic DNA was extracted from the tissues of interest, and then deep sequencing was performed to analyze target genes.

Furthermore, CRISPR-Cas9 with sgRNAs were transduced into TCR-expressing CD8+ T cells to investigate the target genes of long-lived effector T cells 54. The modified T cells were then adoptively transferred into mice bearing B16 melanoma. The sgRNA library was analyzed seven days after the transfer, in which 218 genes were significantly depleted. REGNASE-1 was the most highly increased gene, the functions of which in effector T cells and the anti-tumor immunity were validated. A secondary genome-scale CRISPR library screen was performed to identify the mechanism involved in the REGNASE-1 pathway. BATF was significantly increased in the REGNASE-1-null cells and therefore was the key target of REGNASE-1. By enhancing BATF function and metabolism, REGNASE-1-deficient CD8+ cells were reprogrammed and exhibited an improved response to adoptive cell therapy.

Wang *et al.* used a CRISPRko library containing over 4,500 genes related to tumor development 55. The CRISPR library was transduced into membrane-bound ovalbumin-expressing 4T1 cells, an approach to enhance cellular immune response. Cells with 200-fold sgRNAs were transplanted into the fat pads of normal, nude, and immune-competent (pre-vaccinated with ovalbumin) BALB/c mice. The sgRNA abundance distributions at different levels of host immunity were compared and analyzed. Eventually, E3 ubiquitin ligase Cop1 was identified as a regulator of the macrophage chemoattractant, and its inhibition resulted in increased sensitivity to anti-PD1 treatment and prolonged survival.

In general, the CRISPR library screening approach identified potential key genes in the anti-tumor immunity, but the mechanisms of downstream gene regulation require further characterization. Also, it is currently challenging to search for effective therapeutic drugs against the target as it is not a commonly studied gene due to the randomness of the discovery process.

### Organoids

Some cancers like brain tumors cannot be studied *in vivo* easily due to the absence of a good orthotopic model. Genetically engineered mouse models are a choice, but the genetic features of mice are limited, and the cost is high. Patient-derived xenografts are also not ideal for studying tumor initiation and drug screening. Also, although easy, the use of cancer cell lines is not appropriate for investigations involving cell differentiation, cancer stem cells, and the influence of the tumor microenvironment. In this context, *in vitro* organoid cultures for tumor modeling represent a recent advance often used in drug discovery. However, the CRISPR-based genome editing platform has not been integrated into the organoid models due to technical limitations, such as the need for large cell numbers, heterogeneous growth rates, and low survival rates. Nevertheless, a recent study utilized the CRISPR screen in human intestinal organoids to identify TGF-β resistance genes 56. The investigators performed CRISPR screening with a single organoid sequencing analysis approach, where each surviving clone was individually amplified with different barcoded primers. Since the organoids were grown from single clones, the noise from heterogenous growth rates could be removed entirely.

In another study, Michels *et al.* devised an optimized protocol to apply CRISPR-Cas9 screening in the 3D colorectal cancer (CRC) organoid system 57. They selected TGF-β sensitivity as a positive selection phenotypic trait as this pathway contains several mutated tumor suppressor genes in CRC. The gRNA against TGF-β receptor-2 (TGFBR2) was used as a positive control. The MOI for infecting organoids was titrated by transducing two TGFBR2 gRNA vectors containing GFP or DsRed reporters to be <1. With TGF-β selection, strong signals were observed in 68% of the cells, while a small 5.7% proportion was double-integrated. The pooled library was then transduced to the human colon organoid stably expressing Cas9; cultures were collected after 2 days, 2 weeks, and TGF-β selection for 6.5 weeks, and sgRNAs were analyzed. The group also xenotransplanted the modified organoids into mice models, showing the diverse utilization of CRISPR library screens.

In the studies of brain tumorigenesis, a 3D neoplastic cerebral organoid model was adapted to recapitulate tumor formation 58. Human embryonic stem cells were used to generate embryoid bodies on ultra-low attachment plates and then induced to form neuroepithelial tissues on matrigel. Subsequently, brain tumors were initiated by the transfected plasmids. CRISPR-Cas9 library provided the tumor-suppressor gene mutation, while Sleeping Beauty transposon was inserted for oncogene amplification. The expanded cells were collected, and the gene mutation pattern was analyzed. This model was instrumental in identifying the mutation driver assemblies where the organoids were initiated from the patient-collected pluripotent stem cells.

### Actions of Small Molecules

In the drug discovery process, identifying the cellular targets of the candidate molecules is valuable information. Immunotherapies have improved remarkably in treating multiple cancers, in which chimeric antigen receptor (CAR) T-cell therapy is prominent on B-cell neoplasms. However, primary and acquired resistance is a hurdle 59, and small-molecule inhibitors affect the modulated immune cells. A large-scale drug sensitivity screen was conducted with a genome-scale CRISPR-Cas9 library screen to select potential candidates for enhancing the CAR-T efficacy 60. More than 500 chemical compounds were screened on cytotoxic T cells for function and downstream signaling, and a CRISPR screen was used to investigate the genes responsible for impaired cytotoxicity of therapeutic CAR-T cells. Screening results identified that SMAC mimetics potently sensitized malignant B cells to CAR-T cytotoxicity, and the library screen characterized the mechanism of action of the molecule. The pharmacological effect was mediated through the RIPK1 pathway, indicating the involvement of programmed cell deaths, including necroptosis 61. Hence, the combination of the small molecule profiling and the CRISPR library screen results in a fast and systematic selection of effective compounds with known genetic mechanisms of action. Furthermore, the CRISPR-Cas9 library screen for genome-wide mutations provides a fast and effective way to search for the possible drug resistance genetic variations. Neggers *et al.* demonstrated the beneficial approach of creating localized genetic variations on the gene screening 62. The mutagenesis in candidate genes leading to drug resistance was indicative of gain-of-function screen, while mutations leading to essential protein knockout represent loss-of-function screen. In brief, using CRISPR-Cas9 pooled library is favorable in screening small molecule actions.

## Application of CRISPR-Cas9 Library in Target Discovery and Development of Anti-cancer Drugs

### Essential Genes for Survival

Essential genes or fitness genes are the genes that must be present in surviving cells, and their perturbation would negatively affect cell survival and proliferation 63. A common feature of the cancer genome is chromosomal perturbation, causing lesions in driver genes, passenger genes, and other essential genes 64. Of these, essential genes offer potential therapeutic windows as drug targets in the tumor 65-67. Hart *et al.* improved screening methods for essential genes named as the TKO library screen 5. The TKO library identified more survival essential genes than the first-generation GeCKO screen. The improvements in the sgRNA library design included removing the strong-biased uridines in the last positions of the sgRNA, sequences with high (>70%) or low (<45%) GC contents, and gRNA with more than one potential off-target site on the genome. Up to 12 sgRNAs were designed for each target gene, and 176,500 sgRNAs were targeted on 17,611 coding genes. As discussed in the study, the distribution of the fold-changes on essential genes should not shift remarkably on the graph compared with non-essential genes. In this case, the expression level of the corresponding sgRNAs could be meaningful as a reference of common core fitness genes or cell-dependent pathway-specific essential genes.

The efficiency of CRISPRi and CRISPRko for screening survival-essential genes was studied by Evers et al. The results showed that CRISPRko technology was the most suitable CRISPR-Cas9 platform in lethality screens 68. The various sgRNAs among the same target genes appeared to be consistent. The lowest false-discovery rate among all types of library screens makes it the most promising technology in studying fitness genes 69. Notably, the number of breaks present on the genome by CRISPR-Cas9 editing resulted in an anti-proliferative response unrelated to the gene targets 35. This unpredicted cell toxicity must be handled carefully in the studies of cancer essential genes, as tumor cells tend to have high copy-number alterations in the genome. In summary, the CRISPR-Cas9 library screen aids the identification and characterization of essential genes in tumor cells.

### Identification of Druggable Target Genes

One major obstacle in pharmacological research is the identification of *de novo* druggable targets. The CRISPR-Cas9 library screens can be adopted in different models to facilitate drug discovery. Patel *et al.* developed the 2CT-CRISPR assay system to confirm the critical genes responsible for evasion from the effector T cell response 70. Human CD8+ T cells were isolated and then engineered to be specifically targeted to the HLA-class-1 antigen only. Melanoma cells were transduced with the CRISPRko GeCKOv.2 library and then exposed to the effector T cells. The change in sgRNA levels revealed genes directly related to MHC class 1 antigen processing and elevated unreported genes, such as APLNR, which was later validated for its anti-tumor response of T cells via JAK-STAT signaling.

The CRISPR library screen can quickly and efficiently discover novel drug targets. For example, CMTM6, a previously uncharacterized PD-L1-related protein, was discovered as a potential therapeutic target in the anti-tumor immunotherapy using a similar approach by the CRISPR library screen 71. Also, ferroptosis is a vital cell death mechanism in studying anti-cancer therapies. Using the CRISPR-Cas9 suppression screen, cytochrome P450 oxidoreductase (POR) was essential for ferroptotic cell death in cancer 72. POR knockout induces ferroptosis in various cell lines and is critical for lipid peroxidation. It is a potential druggable target for developing anti-tumor therapies using the ferroptosis approach. In another study applying the CRISPR-Cas9 suppressor screen, peroxisomes were shown to contribute to ferroptosis in renal and ovarian carcinoma cell lines. The results also indicated that inhibition of specific peroxisome component genes lowered the vulnerability of cells to ferroptosis induced by peroxisomes through the synthesis of polyunsaturated ether phospholipids, which could be an essential mediator in the anti-cancer strategy 73.

### Drug Resistance Genes

Mutations are a common cause of acquired drug resistance 74. The information on the resistance-contributing mutations could facilitate the development of detection methods, diagnosis standards, prognosis references, and drug target discovery and design. For example, the drug target of the proteasome inhibitor bortezomib, PSMB5, was mutagenized by a library of 143 sgRNAs incorporating a dCas9-MS2-AIDΔ mutagenesis complex in the human erythroleukemic K562 cell line 75. After transduction and drug selection, mutation frequencies were quantified at every single base pair. The results were validated by separate editing that demonstrated five sites of powerful resistance-induced mutations against bortezomib. Another study investigated the resistance mechanism against immunotherapy in melanoma 76 by transducing a library of 9,872 sgRNAs targeting 2,368 genes into the stable Cas9-expressing B16 cell line. The cells were then transplanted into wild-type and TCRa-/- mice. Apart from the well-known immune evasion PD-L1 and CD47 markers, levels of IFNγ signaling pathway genes targeted by sgRNAs were significantly increased in the immunotherapy group. By analyzing the most depleted genes in the treatment group, PTPN2 was identified to be the cause for resistance to immunotherapy since sgRNAs targeting this gene sensitized the response to immunotherapy. Ptpn2-null tumors were less resistant to the treatment, while tumor suppression was not observed in the TCRa-/- mice. Likewise, a CRISPR-Cas9 screen was performed on an AML-derived cell line MOLM-13 to study the resistance to BCL2 inhibitor venetoclax 77. The cell line was first transduced to express Cas9 and then infected with sgRNA-carrying lentivirus. Cells were selected by puromycin for five days and then followed by venetoclax for 14 days. Sequencing results showed that sgRNAs of TP53, BAX, and proteins in the apoptosis pathway or mitochondrial homeostasis, such as PMAIP1, were enriched most significantly, and their inactivation contributed to drug resistance.

### Non-coding RNAs

With the advances in whole-genome analysis technology, the role of non-coding mutations contributing to tumor development was uncovered. Known mutations in the non-coding regions of the genome can also drive cancer by altering gene expression, transcriptional, post-transcriptional, and epigenetic regulation, regulatory elements, chromatin structure, and regulatory non-coding RNAs 78-80. The non-coding elements in the genome affect the expression of oncogenes and tumor suppressor genes via direct or indirect actions 81-83. As discussed in the previous sections, small-scaled indel cuts or base-pair substitutions by the Cas9 suppression system are not likely to inhibit or elevate the function of the non-coding elements, especially the long non-coding RNAs (lncRNAs). Therefore, the CRISPR-Cas9 system had to be improved for effective functional modulation of non-coding RNAs.

A high-throughput genomic deletion method was proposed in 2016 involving a paired-guide RNA (pgRNA) library 42. The pgRNA strategy is a one-step approach to target two cutting sites for the Cas9 protein with a gap as long as 23kb between the two sites 84. Increasing the number of sgRNA pairs could further enhance the targeting efficiency. It was also suggested that the dual sgRNA system could improve the Cas9-mediated targeting modification efficiency *in vivo* with transmittable on-target and off-target mutations 85. The pgRNA library screening was validated to be specific and more effective than the individual CRISPR-Cas9 knockout, providing another potent tool for studying genome-wide lncRNAs. Although the dual sgRNA system can delete a large fragment of DNA on the site, the targets must be carefully designed to avoid overlapping with other functional non-coding elements in the genome, such as enhancers and miRNAs, or disrupting the introns of other coding genes 86. Also, the screening results provide no information on the molecular mechanism of lncRNAs, requiring further research to understand the downstream interactions 87. The pgRNA library approach can detect loss-of-function, but it is tedious to design and set up and not suitable for gain-of-function screens.

On the other hand, the CRISPRi and CRISPRa approaches are more feasible to disrupt or excite lncRNA expression. The dCas9 protein and a suppression or activation domain could readily modulate gene expression, including the lncRNA level 88. Zhang's group applied the dCas9-VP64 protein with an MS2-p65-HSF1 fusion protein to form a SAM complex, which could upregulate coding genes, intragenic non-coding RNAs, and activate multiple genes concurrently. The activation targets depend on the design of the sgRNA library; for example, more than 10,000 lncRNA TSS were shown to be targeted in a melanoma cell line 89. The screen was performed on the malignant melanoma cell line A375 with BRAF inhibitor vemurafenib as the selection pressure. Sixteen novel target sgRNAs, which were not previously reported, were identified using enrichment ranking analysis. One selected candidate EMICERI, which activated the neighboring genes in a dose-dependent manner, was validated.

Recently, the possibility of targeting sgRNAs on splice sites was suggested. Cutting sites of CRISPR were designed to be within 50-75 bp of the 5' splice donor or the 3' splice acceptor sites flanking the intron sequences. This method of gene perturbation has advantages over CRISPRi and CRISPRa in terms of specificity. The phenotypic effects on target sites in the proximity of essential coding genes have been controversial due to uncertain hits on the precise target or regulatory elements of the corresponding neighbouring gene 41. Therefore, the splice site targeting approach can lower the false positive hits in the library screen as the cutting sites are not next to any coding genes. However, this strategy could only effectively target trans-acting lncRNAs. Therefore, targeting the splice sites prior to the promoters is feasible but not ideal 90.

Besides the non-coding RNAs, the regulatory elements at the epigenomic level are also essential features in oncogene regulation. The CRISPR-Cas9-based epigenomic regulatory element screening (CERES) 91 utilizes dCas9^KRAB^ and dCas9^p300^ proteins to suppress or activate DNase I hypersensitive sites (DHS) 92 by the sgRNA library. Results showed that although the gRNAs did not commonly bring gene expression to more than two-fold change, they were validated by further experiments to confirm the modest regulatory actions. Another study by Fulco *et al.* also reported a similar screen by CRISPR-dCas9^KRAB^ suppression 40. Instead of focusing on DHS, they applied the gRNA targets throughout the genome. Results suggested complicated relationships between genes and enhancers, including multiple genes regulated by one enhancer or more than one enhancer controlling a single gene. There was also evidence of enhancers competing with neighbouring promoters in gene regulation.

With the customized design of sgRNA libraries, complex transcription networks and non-coding regulatory elements on specific or arbitrary genes could be mapped and illustrated. The architecture of the sgRNA library for screening non-coding elements requires extra attention, as there have been reports of off-target effects in the Cas9 library and CRIPSRi/a studies 93. The problem shall be ameliorated by improving sgRNA design strategies 94, producing new variants of Cas9 protein 95, as well as engineering the adaptation requirement of the sgRNAs to CRISPR complex 96.

### Combinatorial Studies

Many biological processes, especially in tumors, are controlled by multiple regulatory genes. A combinatorial genetic screen approach for CRISPR-Cas9 library screens has been adopted to identify the complex associations and interactions between various oncogenes or metabolic functions. The multiplex gene targeting system can reveal potential roles of uncharacterized transcriptomes and functions of untranslated regions 97.

The multiplex genetic modification can be attained by transducing sgRNAs targeting two loci in the same cell. It is also achievable by using CRISPR array encoding for more than one targeting spacers 2, 98. A more advanced design to overcome the problem of multiple transductions would be using the CombiGEM technology 99. This strategy was used to build a multiplex gRNA library by ligating restriction-enzyme-digested sgRNAs into compatible overhangs on the backbones. The effect was tested on ovarian cancer cells, and two sets of drug-target combinations were identified as therapeutic candidates.

Furthermore, combination therapies are commonly used to overcome drug resistance in cancers; however, direct screening for possible combinations is not feasible. Han *et al.* examined a functional genetic interaction (GI) map using a massive parallel pairwise gene knockout 19. More than 21,000 pairs of drug targets were mapped against each other using statistical scoring, and the corresponding lethal drug combinations were identified. More personalized targeted therapies are feasible with the help of this systematic GI network.

Also, a group from the Broad Institute, USA, has reported an innovative methodology in this research field 100. Genetically editing two independent sites became feasible. The researchers could genetically edit two independent sites by ingeniously using two unique Cas9 enzymes in the same transduction. In addition to the typical SpCas9 (isolated from *Streptococcus pyogenes*), they picked the orthogonal SaCas9 protein from *Staphylococcus aureus*. A different set of sgRNAs matching with the SaCas9 was designed with machine learning, and the dual CRISPR system was transduced into different cell lines. This system enabled a simultaneous knockout and overexpression mediated by different CRISPR-Cas9 proteins.

## Technological Advances in CRISPR-Cas9 Screening

### Publicly Accessible Repositories and In Silico Studies

Despite numerous benefits of CRISPR library research, it may not be accessible or affordable for some laboratories. However, ready-to-use sgRNA libraries on some non-profit repository platforms are available to scientists for research purposes with an acknowledgment to the contributor. Another option is to perform secondary analysis on prior CRISPR-Cas9 library screening data to obtain more valuable information. The Bayesian Analysis of Gene EssentiaLity (BAGEL) is a machine learning method for studying gene knockout screening data 101. It offers a greater sensitivity but shorter runtime to identify more fitness genes in library screens. The methodology is easily executed in Python, and therefore it could be widely applied in every laboratory. In addition, sequencing data from multiple CRISPR screens could be analyzed together. A total of 31 library screens against 27 drugs using one cell line were integrated to search for genes responsible for sensitivity or resistance to genotoxic agents 102. Previously unknown DNA repair elements and undetected mechanisms of drug action were discovered from the dataset and demonstrated a valuable learning analysis approach. A project Score (https://score.depmap.sanger.ac.uk/) collects CRISPR-Cas9 whole-genome drop-out screens data across all cell lines to develop a human cancer cell model collection 103. Until July 2021, there were 914 cell lines from 25 tissues collected in the database. The unbiased and systematic framework determines context-specific and human core fitness genes 104. The datasets are analyzed in parallel with the patient genomic data to obtain prioritized candidate cancer drug targets. The Biological General Repository for Interaction Datasets (BioGRID) Open Repository of CRISPR Screens (ORCS) (https://orcs.thebiogrid.org/) is an open repository to store CRISPR screens with comprehensive data. It currently holds 1422 CRISPR screens from 719 cell lines 105, and the sgRNA sequence data were re-formatted and collated to be easily accessible.

### Synergistic Usage of OMICs Platforms with CRISPR Library Screens

Several other cutting-edge molecular biology techniques were adapted together with the genetic editing system to enhance data analysis and depth of the results from the CRISPR library screen. Next-generation sequencing is widely applied in most library screens to determine the remaining sgRNA levels in the selected population compared with the control/wild-type setup. The unique barcode for the sgRNA targets is usually added to the PCR primers and embedded next to the Cas9 target site 106. Recent advances have enabled the integration of single-cell transcriptomics with library screens to map the genetic information that regulates cellular phenotypes 107-109. CRISPR library screen could also be combined with metabolomic analyses, demonstrating resistance to several therapies due to one gene perturbation and identifying the potential target of a metabolic dependency 110. Also, Wang *et al*. utilized integrative omics platforms including proteome, phosphoproteome, and transcriptome to analyze and identify various master regulators 111. CRISPR library screen was subsequently carried out to validate downstream transcription factors and crucial metabolic pathways. A combination of bioinformatics and structural biology is expected to further refine the CRISPR-Cas9 system and facilitate more effective and accurate screening strategies.

### Modified Systems for Enhanced Effectiveness of Genome Engineering

Despite its high efficiency, the unexpected off-target effects of the CRISPR-Cas9-based gene-editing technique limit its wide adoption in clinical studies. Current strategies to reduce undesirable editing include specific directed delivery of CRISPR complex, modification of Cas9, and sgRNA engineering 112. In most settings of pooled library screens, Cas9 endonucleases are modified in different ways to accommodate various experimental needs while not sacrificing accuracy. Two typical Cas9 orthologs are SpCas9 and SaCas9. There are many SpCas9 variants for high-fidelity experiments. SpCas9-HF1, eSpCas9, HypaCas9, and SuperFi-Cas9 are examples of structure-guided engineered proteins in which amino acid residues that contact with the DNA strands are modified 95, 113-115. Another approach for obtaining improved variants is random mutagenesis and end-point selection, examples of which include evoCas9, Sniper-Cas9, and xCas9 96, 112, 116. SaCas9, on the other hand, has a much smaller molecular size than SpCas9 and, due to its easy packaging into viral vectors, is more commonly used in animal models. SaCas9-HF, efSaCas9, and KKH-SaCas9 are alternatives to wild-type SaCas9 for reduced off-target activities while preserving on-target efficiency 117-119.

dCas9 proteins fused with the effector domain are usually adopted for epigenome editing and transcriptional modulation. Furthermore, dual-vector adeno-associated virus (AAV) systems 120, smaller Cas9 orthologues 121, 122, and truncated regulatory elements 122, 123 have been developed to cope with the complicated structure of the editing complex. Although these systems facilitate the integration of epigenetic and transcriptional modulations for *in vivo* applications, many obstacles hinder the *in vivo* genetic modification. Delivery methods such as lentivirus and adenovirus can incorporate large packages. Lentivirus is commonly used in pooled library screens on cell lines or primary cells, while adenovirus is usually adopted in vaccine production or immune therapy research. Most current cancer-related *in vivo* studies involving CRISPR-Cas9 technology transplant modified cell lines because viruses are highly immunogenic in animals 124, 125. AAV systems resulted in less severe host response and cellular damage but elicited the humoral response and T cell activation 126, 127. The *in vivo* CRISPR-Cas9-based genetic modification has not been optimized yet, and nor has the robustness of pooled library screens. The usage of CRISPR-Cas9 library screens in animal models is still in its infancy, and further research is required to enhance the accuracy, potency, and safety.

There are alternatives to Cas9 proteins, such as Cas12, Cas13, Cas14 and Casɸ, but this discussion would be beyond the scope of this review.

## Discussions and Perspectives

The CRISPR-Cas9 genetic editing system is one of the most significant breakthroughs of molecular biology in the twenty-first century. The library screening approach is a remarkable consequence derived from its original function as a ge nome perturbation tool. With machine learning and computing tools, designing a genome-scale sgRNA library with thousands of targets is not a fantasy anymore. Artificial intelligence can now predict if sgRNAs are functional for the CRISPR-Cas9 system 128-131, making it easier to obtain more sophisticated and efficient sgRNA libraries focusing on specific areas of interest rather than screening the entire genome. A well-designed, precise sgRNA library could provide large-scale experimental results for identifying gene functions where the genetic information is obtained from phenotypic selections. Anti-cancer drug discovery is a complicated process that involves the entire spectrum, from determining the genetic mutation causes to identifying the potential drug targets. The research had been like finding a needle in the haystack until the invention of the CRISPR library screen. The pre-clinical stage can be completed faster and more systematically using *in vitro* cell experiments and/or *in vivo* animal models. The CRISPR genetic engineering system enables rapid, versatile, and accurate genome editing. The combination of CRISPR/Cas9 gene-editing tool and omics technologies has enabled genome-wide genetic screen and mutagenesis analysis, revealing the underlying causes of many genetic problems and functions of non-coding elements in the genome. The ultimate goal of eliminating and/or effectively treating cancers appears to be within reach. Oncology research and genome editing have been placed on a fast track, providing hope that soon we would be able to edit our genomes for curing multiple diseases. Along with the exciting and exploding research outputs, we must consider the possible ethical issues that may arise from this ground-breaking advancement. CRISPR technology to modify genetic information in human embryos is prohibited now, and society has not consented to the concept of genetically engineered babies. Also, the potential off-target effect must be eliminated to ensure patient safety for the clinical application of this technology. Modified genes in adoptively transferred cells may lead to inadvertent detrimental results in humans. Therefore, it is imperative to safeguard technological advances for the benefit of humanity.

## Figures and Tables

**Table 1:** Research Milestones for CRISPR-Cas9 Library Screen Development

Name of Library	Type of Screening	Type of Cas9	Study Outline	Model	Research Output	Selection Method	Year [Ref]
Customized sgRNA library	CRISPRko	Cas9	Improving immunotherapy against triple-negative breast cancer	Normal, nude, and immune-competent BALB/c mice	*Cop1* as a target on anti-PD-1 treatment	Puromycin	2022 55
CHyMErA hgRNA pooled library	Multiplex CRISPRko	SpCas9, LbCas12a	Combinatorial pooled genetic screening method	HEK293T/17 cell line	Establishment of guidelines for combinatorial pooled genetic screens (ChyMErA)	Blasticidin, G418	2021 97
SLALOM	CRISPRko	Cas9	Method for enzymatic synthesis of sgRNA library	*E. coli*, zebrafish	Library constructed by a custom sgRNA scaffold sequence	GFP	2021 132
PS4 and EMX1 library	CRISPRko	Cas9 variants, SpCas9, SaCas9, SpCas9 HF1, HypaCas9, HiFi Cas9	Systematic analysis of Cas9 variants in nicking defects	*E. coli* BL21	Multiple Cas9s had varied activities and specificities	/	2021 51
Customized sgRNA library	CRISPRko	Cas9	Screening for tumor drivers in human CRC	colorectal cancer organoid, xenografts	*TGFBR2* as TSG in CRC	Blasticidin, TGFβ	2020 57
Brunello CRISPR library	CRISPRko	Cas9	Identifying TGFβ-mediated resistance genes in CRC	human small intestine 3D organoid	Tumor-suppressive SWI/SNF chromatin remodeling components	Puromycin, TGFβ	2020 56
Customized sgRNAs	CRISPRko	Cas9 variants	Investigating activity and specificity trade-off of Cas9 variants	HEK293T, U-2 OS, K562 cell line	LZ3 Cas9 as high specificity with +1 insertion profile	Puromycin	2020 133
Customized sgRNA library	CRISPRko	Inducible Cas9	Studying the Cas9 toxicity in hPSC	human pluripotent stem cells	Cas9 toxicity was *P53/TP53-*dependent	Puromycin	2018 134
Customized sgRNA library	CRISPRko	hSpCas9	Screening mutations related to brain tumorigenesis	neoplastic cerebral organoid	Identified three combinations inducing abnormal glial growth	Phenotypic screen	2018 58
Brunello CRISPRko	CRISPRko	SpCas9	Optimizing customized sgRNA libraries in genome-wide screens	A375, HT29, MelJuSo cell line	Knockout, interference, and activation CRISPR libraries were optimized in three tumor cell lines	Vemurafenib, selumetinib, trametinib	2018 135
Dolcetto CRISPRi	CRISPRi						
Calabrese CRISPRa	CRISPRa						
Customized sgRNA library	CRISPRi	dCas9-KRAB-MeCP2	Testing the effectiveness of modified Cas9 repressor	HEK293T cell line	KRAB-MeCP2 was an improved Cas9 repressor than KRAB	Various	2018 45
Big Papi	Multiplex CRISPRko	SpCas9, SaCas9, dCas9-VPR	Using combinatorial genetic screening to explore complex gene networks in cancers	A375, HT29, OVCAR8, 786O, A549, Meljuso cell line	Genetic interactions were identified using two orthologous Cas9 enzymes	Puromycin, survival	2018 100
DrugTarget-CDKO library	Multiplex CRISPRko	Cas9	Screening for gene combinations for drug-resistance	K562, GM12892 cell line	*BCL2L1* and MCL1, etc. were responsible for imatinib resistance	Puromycin, ricin	2017 19
Customized sgRNA library	CRISPRi, CRISPRa	dCas9^KRAB^, dCas9^p300^	Screening for functions of regulatory regions by parallel LOF and GOF methods	*In vitro*	Regulatory elements of β-globin locus and *HER2* loci were identified	Survival	2017 91
Dual-gRNA library	Multiplex CRISPRko	Cas9	Mapping cancer genetic networks by combinatorial screens	HeLa, 293T, A549 cell line	Numerous therapeutically relevant interactions were identified	Cell growth	2017 136
GeCKOv.2 library	CRISPRko	Cas9	Profiling genes responsible for T-cell therapy resistance	Mel624, A375 cell line	*APLNR* was identified as the mediator of impaired CD8 T cell	Survival	2017 70
GeCKO library	CRISPRko, CRISPRa	SpCas9, dCas9-VP64-KRABMS2	Optimizing protocol for* in vitro* CRISPRko and CRISPRa	*In vitro* models	Guidelines for screening parameters were provided	/	2017 18
Customized sgRNA library	Multiplex CRISPRko	Cas9	Systematic identification of gene and drug combinations	MDA-MB-231, BxPC-3 cell line	*KDM4C/BRD4* and *KDM6B/BRD4* inhibitors had synergistic efficacy against OC	Zeocin	2016 99
PSMB5 tiling library	Point mutagenesis	dCas9-MS2-AID	Characterizing protein functions by point mutagenesis	K562 cell line	Mutations in bortezomib resistance genes were uncovered	Bortezomib, fluorescence	2016 75
Customized sgRNA library	CRISPRko	Cas9	Characterizing functional enhancers in tumor cells	BJ-RAS, MCF-7, T47D, MDA-MD-231	Several functional enhancer elements mediating *TP53* and *ESR1* were identified	Survival	2016 29
Customized pgRNA library	Paired CRISPRi	Cas9	Screening for functional lncRNAs regulating cancer cell growth	Huh7.5, 22RV1, HeLa cell line	51 targets were identified, and 9 of them were validated	Survival	2016 42
Specialized sgRNA library	CRISPRko, CRISPRi	SpCas9, dCas9-KRAB	Comparing CRISPR with shRNA in lethality screen of tumor cell	RT-112, UM-UC-3 cell line	CRISPR was better than shRNA and CRISPRi based method for identification of essential genes	Puromycin, doxycycline, survival	2016 68
Customized sgRNAs	CRISPRko	Cas9	Investigating amino lipids delivery system of long RNAs	HeLa cell line	Cas9 and DNA editing was sustained 95%	mCherry, luciferase	2016 137
Avana, Asiago library	CRISPRko	Cas9	Optimizing sgRNA design to maximize activity on tumor cell	A375, HT29, MOLM13, BV2 cell line	Rules were set to improve performance	Anti-cancer drugs	2016 138
Customized sgRNAs	CRISPRi	dCas9-KRAB, dCas9-VP64	Generating synthetic transcriptional programs by CRISPR scaffold RNAs	*S. cerevisiae*, HEK293T cell line	Scaffold RNAs could encode target loci and regulatory actions	mCherry	2015 20
Customized sgRNA library	CRISPRko	SpCas9, St1Cas9, SaCas9	Characterizing various functions of Cas9 derivatives	*In vitro*	Cas9 variants were modified to recognize alternative PAM sequences	Bacterial	2015 139
mGeCKOa library	CRISPRko	lentiCas9-EGFP	Identifying genes responsible for tumor growth and metastasis	KPD cell, mouse NSCLC model	Nf2, Pten, Cdkn2a, Trim72, Fga, miR345, or miR-152 KO accelerated tumor metastasis	Primary tumor growth and metastasis	2015 30
Customized sgRNA library	CRISPRa	dCas9-VP64 + MS2-p65-HSF1 (SAM complex)	Screening for BRAF inhibitor resistance genes	A375 cell line	Thirteen genes were identified and validated to confer PLX-4720 resistance	Zeocin, puromycin, PLX-4720	2015 88
Customized sgRNAs	CRISPRko	hSpCas9	Screening for drug targets on acute myeloid leukemia cells	RN2 cell	Six known targets and 19 additional candidates were identified	GFP/mCherry Competition assay	2015 53
TKO library	CRISPRko	Cas9	Identifying cancer fitness genes	A375, RPE1, GBM, DLD1, HeLa, HCT116 cell line	*ANKRD49, ZNF830, CCDC84,* and* RBM48* were identified and validated as cancer-related fitness gene	Survival	2015 5
Customized sgRNAs	CRISPRko, point mutagenesis	Cas9	Identifying chemical compounds that can modulate gene editing by HDR	Mouse embryonic stem cells, Human iPSC	L755507 and Brefeldin A improved while AZT and TFT decreased HDR efficiency	GFP	215 140
Customized sgRNA library	CRISPRko	Cas9	Developing multiplex CRISPR/Cas9 system	*Saccharomyces cerevisiae*	HDR could accurately edit up to five loci without many off-target effects	LEU2 selection	2015 141
Customized sgRNAs	CRISPRko	Cas9, dCas9	Mapping of genome-wide dCas9 binding sites in mESC	mouse embryonic stem cells	Complicated off-target binding was observed, models for Cas9 binding were suggested	/	2014 25
Genome-scale library	CRISPRi, CRISPRa	dCas9-KRAB, dCas9-SunTag	Optimizing CRISPRi and CRISPRa methodology on cancer cells	K562 cell line	Essential genes, TSG, differentiation regulators, and toxin-sensitive genes were identified	Survival	2014 37
Human CRISPR Knockout Pooled Libraries	CRISPRko	hSpCas9	Screening of resistance genes and essential genes on cancer cells	HL60, KBM7 cell line	*TOP2A* and* CDK6* were responsible for etoposide resistance	6-thioguianine, etoposide	2014 10
Customized sgRNA library	CRISPRko	OCT1-Cas9	Functional screening by lentiviral library in human cancer cells	HEK293T, HT1080, HeLa cell line	*PLXNA1, FZD10, PECR, CD81* and* RAB2A* were novel targets involving toxin resistance	diphtheria and chimaeric anthrax	2014 12
Customized sgRNA library	CRISPRko	piggyBac-hCas9	LOF screening for drug resistance genes in mESC	Mouse embryonic stem cells	27 known and four previously unknown genes responsible for drug resistance were identified	*C. septicum* alpha-toxin, 6-thioguanine	2014 11
GeCKO library	CRISPRko	SpCas9	Identifying essential genes and vemurafenib-resistance genes	A375, HUES62 cell line	*NF1, MED12, NF2, CUL3, TADA2B* and* TADA1* were identified as targets	Vemurafenib	2014 9
Evx1 sgRNA library	CRISPRko	SpCas9	Detecting CRISPR-induced mutations	W9.5 mES cell line	37 clones were identified that were disrupted	GFP, survival	2014 106
Specialized sgRNA library	CRISPRko	SpCas9	Optimizing design of sgRNA libraries on cancer cells	MOLM13, NB4, TF1, A375 cell line	Sequence features contributed to Cas9 ability of binding DNA	Puromycin, cell surface marker	2014 142
CRISPR array	CRISPRko	SpCas9	Demonstrating potential uses of CRISPR-Cas9	HEK293FT, N2A cell line	Multiple applications of Cas9 were shown	/	2013 2

**Table 2:** Characteristics of Various CRISPR Screening Technologies

Type of CRISPR screen	Definition	Applications	Advantages	Disadvantages	Examples
CRISPRko Screen	Genome-wide irreversible gene ablation by NHEJ or HDR under the action of CRISPR-Cas9, followed by the screening of resulting phenotypic alternations	To detect the loss of fitness in the cell population, such as reduced viability, drug sensitivity, proliferation, and incapability of migration	- Low noise - Convenient for detecting survival essential genes or fitness genes - Higher sensitivity than previous RNAi platforms - Able to modulate nearly the entire genome, including non-coding components	- Low cutting efficiency - Some off-target effects observed - More sgRNAs are required to ensure effectiveness on each target - Heterogeneous and heterozygote knockouts are observed - Cell toxicity may be induced with increased DSBs in the genome	- Hart *et al.* identified ~2000 fitness genes in five human cancer cell lines, and accurately recapitulated genetic vulnerabilities induced by oncogenes responsive to receptor tyrosine kinases. 5 - Michels *et al.* screened a pan-cancer TSG library in a CTC organoid model and *in vivo* xenograft models with TGFβ resistance as a paradigm. 57 - Wang *et al.* identified E3 ligase *Cop1* as a novel immune target modulating the immune microenvironment in an *in vivo* TNBC model. 55
CRISPRi Screen	Genome-wide reversible gene suppression without perturbating genomic sequence by CRISPR-dCas9, usually adopted with extra regulatory domain, followed by screening of resulting phenotypic alterations	To detect the loss of function in the population; by cooperating with different functional suppression complexes, various precise targets could be obtained	- No perturbation of genetic sequence is needed - No non-target cell toxicity caused - Could interfere precisely with regulatory elements on the genome - Effective in knockdown of lncRNAs	- Vulnerable to sequence variability - Suboptimal in complex TSS regulatory mechanisms - Larger Cas9 complex is needed - Difficult to be packed inside AAV due to its large size, while counterpart lentivirus and adenovirus easily cause host responses	- Gilbert *et al.* established a robust CRISPRi platform, and screened for essential genes responsible for growth and sensitivity to toxin in K562 cells. 37 - Zhu *et al.* utilized a paired-guide RNA approach to screen for human lncRNAs regulating human cancer cell growth, and found nine validated hits. 42 - Klann *et al.* applied the CRISPR-Cas9-based epigenomic regulatory element screening to search for proximal and distal regulatory elements activity. Previously known and unknown elements of β-globin locus and *HER2* were identified. 91
CRISPRa Screen	Genome-wide reversible gene activation without perturbating genomic sequence by CRISPR-dCas9, usually adopted with extra regulatory domain, followed by the screening of resulting phenotypic alterations	By regulating the promoter regions, genes or non-coding elements could be activated or overexpressed. A gain-of-function analysis could obtain information on drug resistance genes or essential proteins.	- No perturbation of genetic sequence is needed - Much better and easier than the previous cDNA library overexpression method - More feasible to excite lncRNA expression by regulating the promoter - Robust *in vivo* activation models	- Vulnerable to sequence variability - Larger Cas9 complex is needed - Difficult to be packed inside AAV due to its large size, while counterpart lentivirus and adenovirus easily cause host responses	Konermann et al. engineered Cas9 activation complex and investigated lincRNA transcripts conferring BRAF inhibitor resistance. 88 - Gilbert *et al.* established a robust CRISPRa platform, and screened for tumor suppressors and developmental transcription factors in cancer cells 37 - Klann *et al.* applied the CRISPR-Cas9-based epigenomic regulatory element screening to examine the activity of proximal and distal regulatory elements. Previously known and unknown elements of β-globin locus and *HER2* were identified. 91
Point mutagenesis	Multiple base-pair mutations or conversion in regions of interest, without causing indels and frameshifts, followed by functional and structural screens.	By using nickase, this CRISPR-based screening approach could screen for point mutations in oncogenes or TSG, characterize protein functions to assist drug target design, and look for mutagenesis causing drug resistance	- High efficiency - Accuracy in editing targets - Obtain extensive information on SNP in a single run	- Base editing complex is enormous - Long foreign genetic materials are introduced into the cells - Limited efficiency in some genomic structures due to spatial hindrance	- Yu* et al.* developed CRISPR-mediated HDR machinery that could cause point mutations, and the action of small molecules could enhance the efficiency. 140 - Hess *et al*. utilized the dCas9 complex with cytidine deaminase attached to create mutagenesis in endogenous targets with limited off-target damage. Known and novel mutations conferring bortezomib resistance were identified. 75
